# Exploring Barriers and Facilitators of Adherence to Artemisinin-Based Combination Therapies for the Treatment of Uncomplicated Malaria in Children in Freetown, Sierra Leone

**DOI:** 10.3390/healthcare9091233

**Published:** 2021-09-18

**Authors:** Kristin Banek, Deborah D. DiLiberto, Emily L. Webb, Samuel Juana Smith, Daniel Chandramohan, Sarah G. Staedke

**Affiliations:** 1 Institute of Global Health and Infectious Diseases, University of North Carolina, Chapel Hill, NC 27514, USA; 2 Department of Clinical Research, London School of Hygiene and Tropical Medicine, London WC1E 7HT, UK; sarah.staedke@lshtm.ac.uk; 3 Global Health Office, Faculty of Health Sciences, McMaster University, Hamilton, ON L8S 4L8, Canada; diliberd@mcmaster.ca; 4 MRC International Statistics and Epidemiology Group, London School of Hygiene and Tropical Medicine, London WC1E 7HT, UK; emily.webb@lshtm.ac.uk; 5 National Malaria Control Programme, Ministry of Health and Sanitation, Freetown, Sierra Leone; samueljuana@yahoo.com; 6 Department of Disease Control, London School of Hygiene and Tropical Medicine, London WC1E 7HT, UK; daniel.chandramohan@lshtm.ac.uk

**Keywords:** malaria, adherence, antimalarial, ACT, artemisinin-based combination therapy, Sierra Leone, qualitative, children

## Abstract

Medication adherence is an essential step in the malaria treatment cascade. We conducted a qualitative study embedded within a randomized controlled trial comparing the adherence to the recommended dosing of two artemisinin-based combination therapies (ACT) to treat uncomplicated malaria in Freetown, Sierra Leone. This study explored the circumstances and factors that influenced caregiver adherence to the ACT prescribed for their child in the trial. In-depth interviews were conducted with 49 caregivers; all interviews were recorded, transcribed, and translated. Transcripts were coded and aggregated into themes, applying a thematic content approach. We identified four key factors that influenced optimal treatment adherence: (1) health system influences, (2) health services, (3) caregivers’ experiences with malaria illness and treatment, and (4) medication characteristics. Specifically, caregivers reported confidence in the health system as facilities were well maintained and care was free. They also felt that health workers provided quality care, leading them to trust the health workers and believe the test results. Ease of medication administration and perceived risk of side effects coupled with caregivers’ prior experience treating malaria influenced how medications were administered. To ensure ACTs achieve maximum effectiveness, consideration of these contextual factors and further development of child-friendly antimalarials are needed.

## 1. Introduction

The malaria treatment cascade has five components contributing to antimalarial effectiveness, including: (1) drug efficacy—the ability to clear parasites from the body and cure patients of malaria; (2) access to treatment; (3) targeting treatment with confirmatory diagnosis; (4) health worker compliance to treatment guidelines, and (5) patient adherence to medication prescribed [1]. The final step, adherence, is considered an essential element for optimal treatment outcomes [2]. The World Health Organization defines adherence as “the extent to which a person’s behaviour—taking medications—corresponds with the agreed recommendations from a health care provider” [3]. Medication-taking behaviours can be influenced by various contextual factors, including: socioeconomic status, health system characteristics, disease symptoms, treatment characteristics, and other patient-related factors [3,4,5].

Antimalarial medications are primarily recommended based on their efficacy. However, other characteristics of the medicines, such as taste, colour, packaging, number of tablets, perceived side effects, and ease of administration, can influence the ultimate effectiveness of the treatment. Previous studies have identified interventions that may improve patient adherence to antimalarial treatment, such as the role of health worker–patient communication [6,7], drug packaging and dosing inserts [8,9,10,11], and community-level interventions aiming to improve awareness around malaria treatment [12]. However, a most effective intervention to improve user adherence has yet to be identified, and achieving optimal adherence may require a combination of approaches [13,14,15].

Although the evidence base on antimalarial adherence is growing, the published literature lacks consensus on factors associated with non-adherence to antimalarial regimens [14,16,17]. An understanding of both individual and contextual factors influencing adherence to antimalarial regimens is needed to guide the development of interventions to improve adherence [14]. Attempts to quantify adherence to prescribed medications should be complemented by an understanding of how and why patients (or caregivers) choose to take or give medications [18,19]. Qualitative research methods provide a way to explore barriers and facilitators influencing medication-taking. However, few qualitative studies have specifically examined adherence to antimalarials and the context in which these medicines are administered [20,21,22,23,24]. This qualitative study explores the circumstances and factors that influenced caregiver adherence to artemisinin-based combination therapies (ACTs) in Freetown, Sierra Leone.

## 2. Materials and Methods

### 2.1. Study Setting

This qualitative study was embedded within a randomized controlled trial that compared the adherence to two ACTs for the treatment of uncomplicated malaria, artemether–lumefantrine (AL) and amodiaquine–artesunate (AQAS), in children under-five at two public health facilities in Freetown, Sierra Leone [25]. The first clinic (Site 1) was located in a densely populated area near the port in the eastern part of Freetown. The second clinic (Site 2) was located in the western part of Freetown. Both health facilities are classified as Community Health Centres by the Ministry of Health and Sanitation-Sierra Leone and primarily provide outpatient, preventative, laboratory, and curative services as well as limited inpatient services for uncomplicated births and childhood illness not considered too severe [26].

### 2.2. Participant Selection

Caregivers with children assigned an adherence outcome (adherent or nonadherent) in the main trial were invited to participate in this qualitative study. Caregivers were purposefully selected to include those who were adherent and nonadherent in each study arm (AL and AQAS) with the aim of enrolling at least 20 participants per study site (10 per study arm). After explaining the purpose of this study, selected participants provided additional written informed consent, including approval to be audio recorded and to be quoted anonymously.

### 2.3. Data Collection and Analysis

In-depth interviews (IDIs) were conducted by the Principal Investigator (PI) or one of two field-workers at each study site using a standardized interview guide. The interviews were conducted one-on-one unless a translator was required or the participant requested another person present. Interviews were conducted in Krio (Sierra Leone national language), although some were conducted in English at the respondent’s request. All interviews were audio-recorded, transcribed, and translated into English when necessary. A summary sheet was prepared for each interview. Demographic, medication, and outcome data collected on the summary sheets were validated by cross-checking the trial case record forms. Analysis of the IDIs was conducted using a thematic content approach [27,28]. The initial analysis involved the PI listening to the interview recordings and reading the English transcripts to ensure familiarity with the data and to confirm the quality of the Krio to English translations. Any discrepancies were reviewed with the study team and the translator. Next, data were coded by identifying common themes and patterns across the data. Coding was initially completed by hand and subsequently uploaded into qualitative data analysis software, MAXQDA Analytics Pro 12 (VERBI Software, Berlin, Germany) [29], to aggregate the codes into themes.

### 2.4. Ethical Considerations

The study was approved by the London School of Hygiene and Tropical Medicine (LSHTM) Research Ethics Committee and the Sierra Leone Ethics and Scientific Review Committee. The full trial protocol including this qualitative study was also registered at ClinicalTrials.gov (NCT01967472).

## 3. Results

### 3.1. Participant Characteristics

The sub-study enrolled 57 caregivers across the two study sites (Site 1 = 27; and Site 2 = 30). Interviews were conducted between September 2013 and January 2014, and a total of 49 caregiver interviews were fully transcribed and translated (Site 1 = 27 and Site 2 = 22). Detailed characteristics of participating caregivers in this sub-study are presented in Table 1. Caregivers were primarily female (>90%) and under the age of 35 (65.4%). More caregivers included in this qualitative study were randomized to receive AL (61.2%). Self-reported non-adherence was a rare event in the main trial (range 3.4–8.5%); as a result, only 9 (18.4%) of caregivers interviewed were classified as nonadherent (5 received AL and 4 received AQAS).

### 3.2. Factors Influencing Adherence

**Figure 1:**
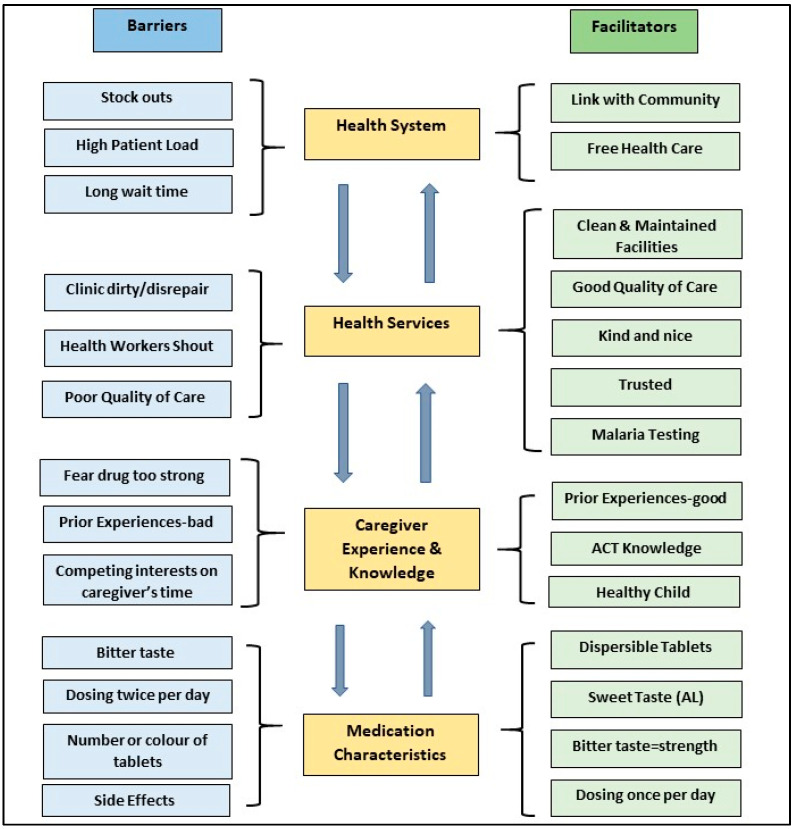
Barriers and facilitators of caregivers to accessing and adhering to ACT treatment for fever in under-five children.

Four domains emerged as key influencers of optimal ACT adherence in this study population: (1) health system influences; (2) health services; (3) caregiver’s experiences with malaria illness and treatment; and (4) medication characteristics (Figure 1).

#### 3.2.1. Domain 1: Health System Influences

##### Access to Free Health Services and Connection to the Health Facility

Caregivers reported that easy access to health care was important and facilitated clinic attendance. Specifically, free services and convenient location were cited by most caregivers as crucial factors that influenced access, the first step on the pathway towards adherence. Respondents reported that feeling connected to the community health centre was also important. In particular, at Site 2, caregivers described feeling loyal to the health centre because they had received antenatal care and delivered their children at that facility; thus, when their children were ill, they took them to that same clinic. Further to this, caregivers from Site 2 considered the study clinic to be the neighbourhood clinic, where people from that community would naturally access health services.

Caregivers reported wanting to seek care at this clinic because the services were perceived to be of good quality and the health facility was well-maintained and clean. Site 1 served the immediate community, plus those seeking care at the nearby children’s hospital referred to outpatient services as their condition did not require tertiary care. As a result, Site 1 had a broader catchment community.

##### Health System Challenges

Caregivers described that delays in receiving the blood test results or treatment, in addition to regular stock-outs, were barriers to accessing care. While the study was ongoing, the patient load was high at both clinics, particularly on immunization days and therapeutic feeding days. The high patient load translated into long wait times for registration and lab tests, and contributed to health workers having less time for consultations. Clinic observations during the trial show that the time constraints directly impacted the quality of the patient–provider interactions (unpublished). The high patient volume also put a strain on resources and drug stocks, ultimately impacting prescribing practices.

*“If there is less number of people, I will not wait long, but if the people are many, I will wait for a long time.”*

(S2-04, female caregiver, nonadherent, AQAS)

*“They don’t give enough medications … I normally decide to go to buy the medicines [at the pharmacy].”*

(S2-16, female caregiver, nonadherent, AL)

##### Consequences of Stock-Outs

Before the study, both health facilities experienced regular stock-outs of ACTs, specifically infant and child doses. As a result, health workers resorted to using adult doses of AQAS to meet the paediatric ACT needs; AQAS adult tablets were cut in half to ensure children were given the correct weight-based dose. This practice became part of the caregivers’ experience with ACTs and impacted how they used all malaria medications subsequently (i.e., cutting tablets in half even if they were given the correct dose or a different antimalarial).

*“I will go by the instruction of the doctor. They will tell me, give him half in the morning and half in the evening. That’s what I do.”*

(S2-05, female caregiver, nonadherent, AQAS)

Some caregivers had difficulty differentiating paediatric and adult formulations of ACTs, which may have resulted from the common practice of cutting and separating blister-packs of adult doses to administer to children. One respondent specifically stated that the co-packaged AQ + AS was for adults and not for children, thus she did not give it to her child.

*“I have never used it [AQ + AS co-pack] with my children because that one is not meant for children. It is for grown-up people, because even myself, I used to use Artesunate.”*

(S1-16, female caregiver, nonadherent, AL)

#### 3.2.2. Domain 2: Health Services

##### Quality Health Services

Caregivers reported that the quality of health services was very important. Quality care was described as having a clean and well-maintained facility, being treated ‘well’ (i.e., courteously) by health facility staff, availability of testing to confirm malaria, and receiving good medications. Caregivers reported wanting to go to the clinic because the services were good and the health facility was well maintained and clean. This was particularly noted by caregivers attending Site 2.

*“Well, the first reason I like taking my child to [that clinic] is that it is located in my community. It is not situated far off. Second, the hospital [clinic] is clean, the nurses are caring, and they talk with us patients nicely. That is why I like taking my child there, whenever my children are affected by fever.”*

(S2-12, female caregiver, adherent, AQAS)

*“The medicines that were supplied to us were very good. They will never come to tell us lies. The doctors and nurses that are there are specialized, and they have never falsely diagnosed any sickness on my child, so I believe them.”*

(S2-19, female caregiver, adherent, AL)

##### The Crucial Role of Health Workers

In general, caregivers felt that health workers at the two study clinics were knowledgeable, honest, and helpful, with those showing kindness to children and caregivers deemed more competent. Almost universally, caregivers deferred to health facility staff as the best person to diagnose their child’s illness and to guide them on fever management.

*“Doctors and nurses will advise me what is wrong with my child.”*

(S1-08, female caregiver, adherent, AQAS)

*“These quack doctors [medicine peddlers] were never trained, they too only buy and sell. But as for the nurses and doctors, they went through training. And they thus know the correct medicines to prescribe.”*

(S2-12, female caregiver, adherent, AQAS)

Furthermore, caregivers reported that their interactions with providers during consultations were positive and that health workers took the time to examine their children and explain how to administer ACTs carefully.

*“He [the health worker] observed my child, diagnosed her. He looked inside her mouth. He looked at her eyes, and he even looked in her ears. And then he had to prescribe medicine for me. After prescribing the medicine, he showed me how to administer the medicine.”*

(S2-23, female caregiver, adherent, AL)

##### Testing for Malaria (Targeting Treatment)

Caregivers reported that diagnostic testing for malaria and receiving a confirmatory diagnosis was necessary, and they associated the presence of testing with better service and more reliable care.

*“For me, it is especially for the good treatment and the test that they do to determine the illness affecting the child.”*

(S2-25, female caregiver, adherent, AL)

*The test is very important. Because sometimes some children will only be affected with fever and nothing else … but if you do a medical test, the test will be able to prove if she has malaria.”*

(S2-28, female caregiver, adherent, AL)

*“… it is the test that they conduct that would tell that it is malaria affecting her.”*

(S1-13, female caregiver, adherent, AL)

#### 3.2.3. Domain 3: Caregiver’s Experience with Malaria Illness and Treatment

##### Medication Knowledge

Very few caregivers used the biomedical names of the antimalarial medications given during the study or received previously. There were mentions of chloroquine and quinine, but in general, the majority of caregivers used the term “malaria treatments” or “malaria medicines.” When caregivers were shown a sample or told the commercial brand-name of the drug (i.e., Lokmol for AL, which has radio advertisements), they quickly identified the malaria medications. Other names used were “government medicines,” and a few used the acronym “ACTs.” Often only the colour or the number of tablets were given to identify the medication. Despite the lacking knowledge of the biomedical names of ACTs, caregivers were familiar with the treatment; they could describe the appearance of each medication and their experience with it.

Almost all of the caregivers interviewed could correctly describe how to administer ACT. Caregivers were quick to respond when asked about directions for administering AQAS, “*one today, one tomorrow and one next tomorrow [the day after tomorrow]*.” Caregivers also knew that AL was to be given twice a day for three days and knew the correct number of tablets for the infant dose (one tablet) and child dose (two tablets).

##### Caregiver Prior Experiences

The caregivers’ interaction with the health system appeared to have an impact on positive health behaviours. Specifically, previous positive experiences at the health facility helped build the caregivers’ trust in the health workers and the medication.

*“Even myself as an adult, when I do go there [to the clinic] for treatments; they would treat me well and ensured that I went through all the procedures that I should until I meet the CHO [community health officer] … So that is why I like them. They are really doing their job.”*

(S2-02, male caregiver, adherent, AL)

Additionally, caregivers’ personal experience with antimalarial medications and their own response to treatment influenced how they administered medications to their children. Specifically, medications that were felt to work quickly, resulting in rapid improvement, were considered ‘strong’ or more effective (Table 2). Caregivers also reported that medications were superior if they provided a durable cure, meaning that repeated visits to the health facility could be avoided, as well as contributing to their child’s immediate recovery and longer-term health (Table 2).

##### Caregiver Fears

The caregiver’s own fears resulting from previous experiences with malaria episodes and antimalarial treatment (both their own and their child’s) were critical influences on their adherence behaviours. Caregivers were particularly concerned for their children’s health and expressed fear when their child was sick with malaria, “I’m badly afraid of fever” (S1-07, female caregiver, adherent, AL). Most knew that malaria could be fatal, understood the importance of seeking treatment early, and were genuinely concerned with taking the correct steps to protect their children. They also understood the importance of completing the medication course, and that without proper treatment, their child’s condition would not improve.

*“Malaria is a bad killer disease. If immediate treatment is not taken, it kills quickly. The child will not be cured because he has not been given the complete dosage.”*

(S1-05, female caregiver, adherent, AQAS)

*“As long as you follow the doctor’s instructions and take all of the dosages, you will be cured by it.”*

(S2-12, female caregiver, adherent, AQAS)

*“It [AQAS] is also a good cure for malaria…But once you are done taking the dose, you feel comfortable because you have taken the correct malaria treatment.”*

(S1-24, female caregiver, adherent, AQAS)

##### Caregiver Time

Caregivers reported that when their child displayed symptoms of illness, such as not eating or playing, they were more vigilant and ensured they administered the medication correctly to guarantee their child’s improvement. However, once their child became more active, started to play and eat again, they believed the “emergency” had passed. At this point, caregivers reported they shifted their focus back to their normal daily routine, and some reported forgetting to administer the medication to their child as prescribed. Although most caregivers appeared to understand the importance of administering the complete course of treatment, they said they might not adhere strictly to the recommended dosing frequency or timing.

*“When there is an improvement in the health of the child, I would forget to administer it to the exact time, but before the end of the day, I would remember and give it back to him. I would make sure that I gave him all the treatment even if it isn’t administered at the correct time. I give it all.”*

(S2-04, female caregiver, nonadherent, AQAS)

##### Treatment Administration

Methods of administrating ACTs varied. For AQAS, almost all caregivers reported grinding the tablets, adding to water, and dispensing either with a spoon or a dropper. All caregivers mentioned that they bought bottled water to administer the ACTs. Generally, AL was dissolved in water and given to the child to drink; the simplicity of administering the dispersible formulation contributed to AL’s appeal.

*“Why I said it is good? I said it is good because it is a medicine but does not resemble … it is syrup like when you mix it—the flavour. It has a very nice flavour. When I gave it to my child, she would drink it.”*

(S2-16, female, nonadherent, AL)

One mother of an older child reported that her child swallowed the tablets whole to avoid the bitter taste of AQAS. Many caregivers mentioned that their child actually chewed the tablets and drank the water afterward. This was most common for AL, although a few caregivers mentioned that their child chewed AQAS.

*“The child has the urge to take it [AL]. At times he takes it [AL] without drinking water. He can just chew it like that, in whole.”*

(S2-19, female caregiver, adherent, AL)

*“Whenever I gave it [AL] to her, she would chew it and drink water…she was chewing every bit of it because she is a child that likes chewing medicines.”*

(S2-28, female caregiver, adherent, AL)

*“This medicine is nice; it has a nice smell. It is nice. It has a strawberry smell. That is why I like it. Whenever I gave it to my child, he would just chew it. He likes it too [very] much.”*

(S2-23, female caregiver, adherent, AL)

Caregivers had different practices regarding the administration of medications with food. Some caregivers felt that giving the medication with food helped to avoid vomiting and other side effects. In contrast, others felt that administering the medication without food was preferable to prevent adverse events.

*“Once the child can eat, he should eat enough because that medicine too [ACT] can cause the child to become weak [from the medication] if he does not eat enough.”*

(S2-22, female caregiver, adherent, AL)

One caregiver mentioned that the quantity of food and timing of eating in relation to the administration of medications were essential to avoid vomiting.

*“No, he doesn’t vomit. This is because after I have fed him, I wait for some time before giving him the medicine [AQAS]. Also, when I give him the medicine, I don’t give him too much water to drink. If I give him too much water, he will vomit. I make sure that I put small water and I grind it very good in that small water and then put it in his mouth, so it all goes down and no vomiting.”*

(S2-04, female caregiver, nonadherent, AQAS)

#### 3.2.4. Domain 4: Medication Characteristics and Administration

##### Medication Taste

Caregivers reported that medicine characteristics (in particular taste) were important factors influencing the acceptability of treatment and ease of administration. Respondents described the ACTs as “*sweet*” or “*bitter*.” “*Sweet*” was seen as a positive trait. Caregivers reported that “*sweet*” medicines were easier to administer to children.

*“It [AL] tastes sweet and also tastes nice.”*

(S2-13, female caregiver, adherent, AQAS)

*“Because this one is not bitter like that yellow and white one. For this one, it has that flavour, that orange-like flavour.”*

(S1-15, female caregiver, adherent, AL)

*“Bitter”* medicines, on the other hand, were viewed both positively and negatively by caregivers. Some caregivers cited bitter taste as a barrier to administration, while others reported that the bitter taste implied that the drug was potent, and thus more effective.

*“No, she was not willing, but she didn’t vomit it also. She didn’t like it because it was bitter.”*

(S1-02, female caregiver, adherent, AQAS)

*“Only that they do vomit because it seems to be very bitter.”*

(S1-01, female caregiver, adherent, AQAS)

*“It is very bitter, but it is for your well bodi [health].”*

(S2-0, female caregiver, nonadherent, AQAS)

##### Tablet Appearance and Medication Packaging

Caregivers associated yellow antimalarial tablets with weakness, generally referring to amodiaquine (which caregivers identified by pointing to the medication during the interview). Interestingly, AL is also yellow, but is packaged in a distinct blister-pack card and has a sweet smell and taste; yellow AL tablets were not mistaken for amodiaquine. Most other tablets commonly used by caregivers (i.e., acetaminophen tablets commonly called “Panadol”) are white and more difficult to identify outside of a distinct package.

**Figure 2:**
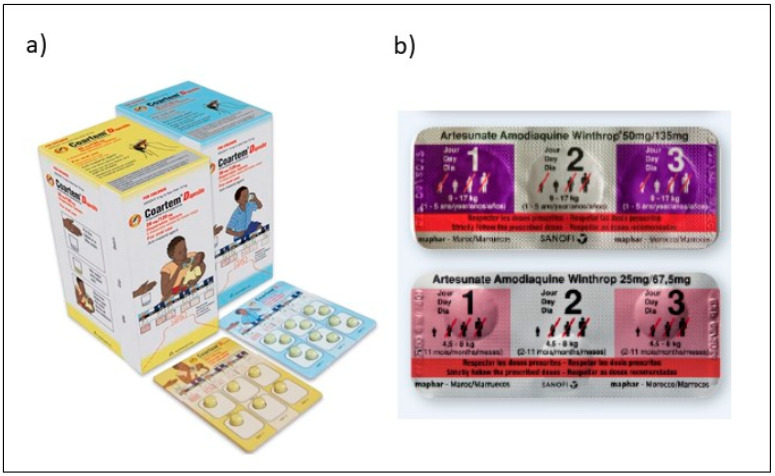
ACT Packaging for children (**a**) Coartem^®^ artemether–lumefantrine Dispersible packaging [30]; (**b**) Winthrop^®^ Fixed-dose artesunate–amodiaquine packaging [31].

Caregivers often identified ACTs not only by their colour, but also by their pharmaceutical packaging (Figure 2). ACT packaging served to identify the antimalarial drugs and as a reminder of how to administer the treatment dose. This was particularly true for AL. More caregivers commented that the packaging for AL was attractive and helpful. AL’s package is colourful, contains pictures to aide treatment administration, and is physically more durable than the packaging of AQAS. Furthermore, respondents mentioned that they liked to keep the AL package to serve as a reminder if they wanted to access this “new” malaria treatment from a pharmacy in the future. Little was mentioned about the details of the packaging for AQAS, but its pharmaceutical packaging indicated that it was a malaria medicine. The exception to these positive associations was when there were stock-outs of paediatric ACTs and when tablets were removed from their blister-packs, cut in half, wrapped in paper, and dispensed to the caregivers who then relied on other characteristics of the medications to distinguish the tablets.

##### Number of Tablets

Caregivers equated the number of tablets with the strength of the treatment. While some caregivers felt that more tablets were ‘*good*,’ while others were reluctant to administer multiple tablets to their child, fearing that too many tablets would be too ‘*strong*,’ and thus harmful, to the child. One caregiver reported giving more tablets at the beginning of treatment, as her child was very sick, while another respondent associated multiple tablets with an increased risk of side-effects, such as weakness following treatment. Caregivers also commented on the difficulty of administering multiple tablets required for AL treatment.

*“Well, the reason for giving her two tablets was because she was seriously sick at that night; she was very weak at that night. The following morning, I only gave her one tablet. And the other day, I continued giving her two tabs again.”*

(S2-17, female caregiver, nonadherent, AL)

*“Even though they said I should administer it two each time, although I was afraid to give two at a time because I thought it would be strong for her, but those two at a time that I was giving to her, I thank God for that. Those two to three days she became very playful once again.”*

(S2-25, female caregiver, adherent, AL)

##### Potential Side Effects

The perception of potential side effects influenced how some caregivers administered ACTs, both in terms of timing and dosing of the medication. One caregiver reported that they would complete the entire dose prescribed, but would stretch out the treatment over four or more days, not delivering it within the recommended three days. Other caregivers reported cutting the AQAS tablets in half or only giving one AL tablet (rather than the two tablets prescribed per dose) to avoid overdosing their children. Caregivers also altered the administration of treatment based on side effects and symptoms related to malaria, including weakness, vomiting, lack of appetite, and constipation. Vomiting, in particular, was cited as a reason to stop giving their children the medicines.

*“When I gave him the malaria tablets [type not specified], he vomited twice. I didn’t give the medicine again. I didn’t give him at all. I went to the doctor.”*

(S2-15, female caregiver, adherent, AL)

## 4. Discussion

Quantifying adherence to prescribed medications should be complemented by an understanding of how and why patients choose to take their medications [18,19]. The primary objective of this qualitative study was to explore the circumstances and factors that influenced caregiver adherence to antimalarials in Freetown, Sierra Leone, within a randomized controlled trial comparing the treatment adherence of two artemisinin-based combination therapies (ACT) for the treatment of uncomplicated malaria. This study identified four key domains along the treatment effectiveness pathway that influenced adherence to ACTs in this study population: health system factors, health services, caregiver experience, and medication characteristics. Our findings are consistent with prior research assessing longer-term therapies [3] as well as non-ACT antimalarials [8,32,33], suggesting that a range of individual and contextual factors influence treatment adherence.

The literature suggests that a collaborative relationship to care may improve adherence [34]. Moreover, having a trusting patient–provider relationship or having very high trust in primary providers has been shown to promote cooperation, access, utilization of services, and adherence [35,36,37]. In this study, malaria diagnostic testing appears to be widely accepted and builds the relationship between provider and caregiver. The extent to which access to free commodities (drugs and diagnostics) influences this relationship is unknown. However, responses from the caregivers suggest that they relied on the health workers to diagnose their children (as the clinic has the tests) and to tell them how to manage the illness. Further understanding of the importance and value of patient and caregiver trust in health systems is needed, as it may impact access and overall service delivery, as well as treatment adherence [35,36,37]. We found that caregivers in this population were generally satisfied with their care, which has been demonstrated elsewhere to improve the recall of treatment advice and thus adherence [38]. Furthermore, there was a great emphasis by participants in this study on the high quality of the care received at the study sites, in particular on how kind the health workers were to them. Sympathetic health workers, and positive patient–provider communication and interactions have been noted to improve adherence for chronic illnesses [36,39] as well as for the treatment of malaria [6].

We found that caregivers built a knowledge base about malaria treatment through their previous experience with treatment. Caregivers in this study population recognized the malaria medication packages, drawing from their prior experience using the medications to treat themselves or their children, even if they were not familiar with their biomedical names. This experience (both good and bad) influenced their opinion of the medication, how best to administer it (crushing, chewing, dissolving, with/without food, etc.), and ultimately, if they or their child were able to complete the treatment. Moreover, caregivers were more attuned to treatment administration when their child’s symptoms were severe, and became more relaxed once the child started to eat and play again [40]. Caregivers in this study reported being more vigilant when their child was sick because they were afraid for their child’s health and life. Similar to the findings from White et al., as the parasites were cleared and symptoms resolved, the caregivers in this study would transition out of this emergency mode into a more relaxed state and discontinue or forget to administer the treatment [41]. A Kenyan study had comparable results, finding that lack of fever or severe disease was associated with non-adherence to ACTs [42]. Likewise, it has also been suggested that caregivers discontinued other antimalarials if symptoms resolved prior to completing the treatment, including the three-day treatment of chloroquine [8,18,43], three-day treatment with amodiaquine/sulphadoxine–pyrimethamine [40], or the longer seven-day quinine treatment [43]. As incomplete treatment can lead to poor patient outcomes, higher treatment costs, and even resistance [14], educating patients and caregivers to complete treatment despite not feeling ill is essential for acute illness, and also in the context of malaria chemoprevention.

Previous studies have demonstrated that users prefer certain antimalarial drugs, particularly those with simple dosing schedules [44,45,46]. Likewise, paediatric medication adherence is known to be influenced by taste and formulation [34,47,48]. Caregivers in this study suggested that flavoured medications that were easy to drink were easier to administer to children. Simple dosing and administration are likely key factors influencing paediatric ACT adherence. While the newer ACTs have included many desirable medication characteristics such as simple dosing schedule, flavouring, and ease of administration [49], they still require water for administration. Many caregivers reported they used a commercial brand of water for medication administration, indicating that they purchased water as part of the treatment. While urban populations may have access to bottled water, more impoverished or remote populations may not. Access to clean water for taking medications is lacking in many resource-poor countries (only 68% in Sub-Saharan Africa [50] and even lower for poorer households [51]). Caregivers in this study reported that their children would chew tablets rather than have them be dispersed in water. A chewable option might save costs and improve medication adherence.

The data presented here suggest that a more comprehensive approach should be taken when designing interventions to improve adherence. The review by Yeung and White found that adherence to antimalarials was higher when “interventions focusing on provider knowledge and behaviour, drug packaging and provision of correct dosage” were implemented [14]. This echoes earlier research that highlighted the importance of patient education, provider communication, and community sensitization on the treatment of acute malaria illness [6,9,12]. Similarly, consideration of the complexity of the diagnosis and treatment process and the role that the different actors’ (both health workers and patients) behaviours play in effective malaria treatment is essential [32,52].

This study had several limitations. First, IDIs took place after the main trial enrolment period (i.e., after the final follow-up interview to measure adherence); thus, caregivers may have had difficulty differentiating between the most recent treatment and those previously received. Second, although we attempted to select participant caregivers based on their outcome classification and study arm, this did not work in practice. Selection criteria for the qualitative study were based on self-reported treatment completion (adherence), which was relatively high in this population (>90%), and limited the number of nonadherent participants available. Furthermore, those willing to participate were mainly from the adherent group rather than from the nonadherent group. Third, the proportion of male caregivers was low for this study. This is not unexpected as females are the primary caregivers for children under five in Sierra Leone. As the primary caregiver, they would also be responsible for medication administration, thus in the best position to describe medicine-taking behaviours. While these limitations may have contributed to some bias in our results, the consistencies in the stories provided across the study sites, participants, and treatment arms suggest that the data collected are reliable. Finally, participant bias and social desirability bias may have occurred during this study, with caregivers providing answers they felt would be acceptable to the researchers or out of a desire to please the interviewer. Bias was mitigated by using a conversational approach in which the caregiver told the story of how the medications were administered. A longer-term ethnographical approach may have built more rapport over time and yielded richer data. However, as this was a sub-study focusing on caregivers’ experiences and perceptions and not strictly an anthropological study, interviews were the preferred data collection method.

## 5. Conclusions

Adherence behaviours have multiple influences. This study identified four key domains that influence adherence to antimalarials in the context of a randomized trial: health system factors, health services, caregiver experience, and medication characteristics. Given the complexity of adherence, holistic interventions that target multiple domains are needed to ensure that ACTs achieve their maximum effectiveness. Furthermore, the continued development of newer antimalarials that are easier to administer (i.e., dispersible or chewable formulations) may improve treatment adherence in children.

## Figures and Tables

**Table 1:** Characteristics of IDI participants.

	Site 1 n (%)	Site 2 n (%)	Total n (%)
Number of IDIs Conducted	27	30	57
Excluded ^1^	0	8	8 (14.0%)
Total number of IDIs analysed	27	22	49 (86.0%)
Caregiver Gender			
Female	24 (88.9%)	21 (95.5%)	45 (91.8%)
Male	3 (11.1%)	1 (4.5%)	4 (8.2%)
Caregiver Age ^2^			
<25	8 (29.6%)	8 (36.4%)	16 (32.7%)
25–34	11 (40.7%)	5 (22.7%)	16 (32.7%)
35–44	3 (11.1%)	5 (22.7%)	8 (16.3%)
45 +	3 (11.1%)	3 (13.6%)	6 (12.2%)
Child Age			
6–23 months	15 (55.6%)	9 (40.9%)	24 (47.1%)
24–59 months	12 (44.4%)	13 (68.2%)	27 (52.9%)
ACT received during the trial			
AQAS	10 (37.0%)	9 (40.9%)	19 (37.3%)
AL	17 (63.0%)	13 (59.1%)	30 (61.2%)
Treatment uptake			
Non-adherent	1 (3.7%)	8 (36.4%)	9 (18.4%)
Adherent	26 (96.3%)	14 (63.6%)	40 (81.6%)

^1^ Eight participants were excluded. Reasons for exclusion: 1 = no recording, 4 = consent not verified; 3 = excluded from RCT after recruitment, so not eligible for the qualitative study. ^2^ Three caregivers did not provide their age (Site 1 = 2; Site 2 = 1).

**Table 2:** Quotes about prompt recovery.

“This medicine is good … whenever you give a medicine to the child, and they recover you must know it is good. I think it is good.” (S1-13, female caregiver, adherent, AL)
“At the time I was sick I drank [malaria] medicine I immediately recovered. So when my children fell sick too, I was giving the [malaria] medicine to them too.”(S1-08, female caregiver, adherent, AQAS)
“Well my child had a severe fever, but when I gave it [AL] to her, I discovered that she recovered. (S1-13, female caregiver, adherent, AL)
“Because upon using it [AQAS] my child recuperated properly. I didn’t have any problem [sickness] again with her up till now.” (S1-14, female caregiver, adherent, AL)

## Data Availability

Data are available from the corresponding author, upon reasonable request.
